# AAV9 gene transfer of cMyBPC N-terminal domains ameliorates cardiomyopathy in cMyBPC-deficient mice

**DOI:** 10.1172/jci.insight.130182

**Published:** 2020-09-03

**Authors:** Jiayang Li, Ranganath Mamidi, Chang Yoon Doh, Joshua B. Holmes, Nikhil Bharambe, Rajesh Ramachandran, Julian E. Stelzer

**Affiliations:** Department of Physiology, School of Medicine, Case Western Reserve University, Cleveland, Ohio, USA.

**Keywords:** Cardiology, Gene therapy, Heart failure

## Abstract

Decreased cardiac myosin-binding protein C (cMyBPC) expression due to inheritable mutations is thought to contribute to the hypertrophic cardiomyopathy (HCM) phenotype, suggesting that increasing cMyBPC content is of therapeutic benefit. In vitro assays show that cMyBPC N-terminal domains (NTDs) contain structural elements necessary and sufficient to modulate actomyosin interactions, but it is unknown if they can regulate in vivo myocardial function. To test whether NTDs can recapitulate the effects of full-length (FL) cMyBPC in rescuing cardiac function in a cMyBPC-null mouse model of HCM, we assessed the efficacy of AAV9 gene transfer of a cMyBPC NTD that contained domains C0C2 and compared its therapeutic potential with AAV9-FL gene replacement. AAV9 vectors were administered systemically at neonatal day 1, when early-onset disease phenotypes begin to manifest. A comprehensive analysis of in vivo and in vitro function was performed following cMyBPC gene transfer. Our results show that a systemic injection of AAV9-C0C2 significantly improved cardiac function (e.g., 52.24 ± 1.69 ejection fraction in the C0C2-treated group compared with 40.07 ± 1.97 in the control cMyBPC^–/–^ group, *P* < 0.05) and reduced the histopathologic signs of cardiomyopathy. Furthermore, C0C2 significantly slowed and normalized the accelerated cross-bridge kinetics found in cMyBPC^–/–^ control myocardium, as evidenced by a 32.41% decrease in the rate of cross-bridge detachment (*k*_rel_). Results indicate that C0C2 can rescue biomechanical defects of cMyBPC deficiency and that the NTD may be a target region for therapeutic myofilament kinetic manipulation.

## Abstract 2

Cardiac function improves following AAV9-mediated delivery of the C0C2 domains of cardiac myosin-binding protein C in a mouse model of hypertrophic cardiomyopathy.

## Introduction

Heart failure (HF) is frequently a result of inherited cardiomyopathies, which are caused by genetic mutations that disrupt cardiomyocyte function (1, 2). Among the inherited cardiomyopathies, hypertrophic cardiomyopathy (HCM) has been linked to mutations found in over 20 genes but has shown substantial variance in phenotypic presentation and disease severity (2). Since mutations in the *MYBPC3* gene, encoding cardiac myosin-binding protein C (cMyBPC), are some of the most frequently implicated in HCM (3), understanding cMyBPC’s role in regulating myocardial contractile function has gained increasing interest in recent years. A great majority of these mutations involve truncation that lead to decreased cMyBPC expression (4–6). Furthermore, in rare but severe cases, human patients with homozygous or heterozygous compound MYBPC3 mutations lead to rapid neonatal HF within the first year of life (7, 8). Thus, the idea of increasing cMyBPC expression was proposed as a curative strategy and has since produced promising results in small animal studies (9). A similar strategy of increasing full-length (FL) cMyBPC expression using a neonatal homozygous cMyBPC-knockin mouse model has also been investigated with encouraging results (10). The striking suppression of pathogenic remodeling in these studies highlights cMyBPC’s indispensable role in regulating sarcomere contractile function.

**Figure 1:**
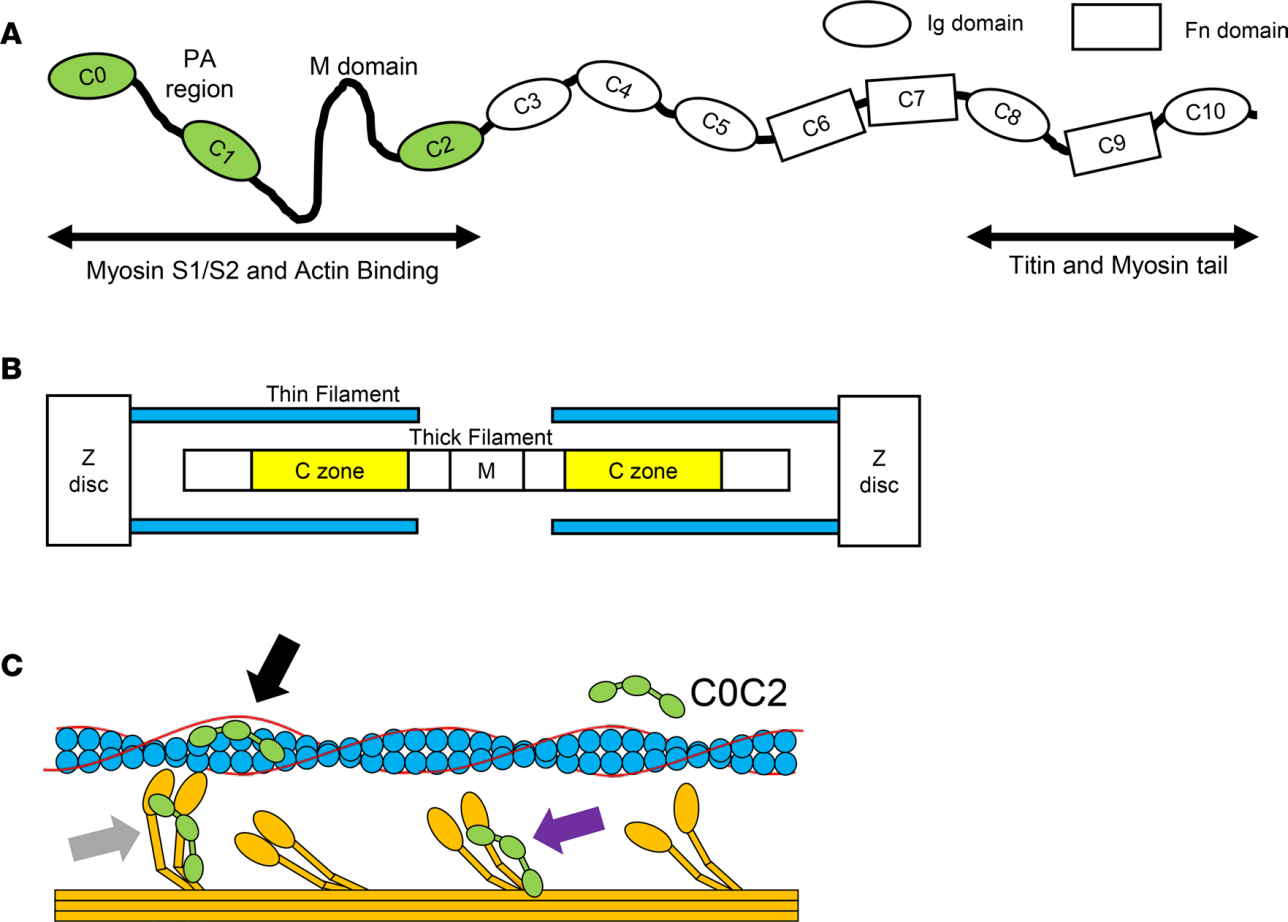
Structure and function of cMyBPC and C0C2. (**A**) Schematic representation of full-length cardiac myosin-binding protein C (cMyBPC) showing N-terminal (C0C2) regulatory domains and C-terminal (C8C10) anchoring domains. (**B**) Illustrated representation of myofilament organization with yellow bars highlighting the C zones where endogenous cMyBPC localize. (**C**) Illustration of C0C2 N-terminal domains showing possible interactions with thin filament (blue), tropomyosin (red), and thick filament (orange) that have been demonstrated in in vitro assays. It has been proposed that C0C2 can directly bind to the thin filament and displace tropomyosin toward an “open” state, exposing adjacent myosin binding sites to allow cross-bridge formation and effectively activating the thin filament (black arrow) (18). C0C2 has also been implicated in direct binding to the S1 and S2 regions of myosin (purple arrow), shifting some myosin heads into “super-relaxed” states toward the thick filament backbone and rendering them unavailable for cross-bridge formation (20, 72). A reduction in cMyBPC expression has been proposed to cause hypercontractility in HCM by freeing these myosin heads out of the “sequestered” state. Another proposed mechanism is the binding of cMyBPC to myosin heads once they are in the strongly bound state (gray arrow). This has been suggested to provide biomechanical stability to the cross-bridge and increase cross-bridge lifetime (29).

cMyBPC is composed of 11 immunoglobulin-like and fibronectin-like domains, numbered C0–C10 (Figure 1A). It is now understood that the C-terminal domains (CTDs) are responsible for tightly anchoring cMyBPC to the thick filament (11). These anchoring sites dictate the stoichiometric distribution of cMyBPC within the sarcomere, restricting cMyBPC to the C zone of the thick filament, with 9–11 stripes regularly spaced ~42 nm apart (12) (Figure 1B). In contrast, in vitro studies have shown that the N-terminal domains (NTDs) of cMyBPC contain the regulatory regions that are both necessary and sufficient to modulate sarcomeric contractility. Early studies of NTDs demonstrated that C1 and C2 domains increased skinned myocyte Ca^2+^ sensitivity in a dose-dependent manner (13). Other studies have since demonstrated both activating and inhibitory effects of NTDs using various in vitro experimental approaches (14–16). The apparent paradox of this dual activating and inhibitory effect has provided mechanistic insight into how cMyBPC functions in the intact cardiomyocyte.

Several studies have confirmed the initial findings in other variations of NTDs, which suggest that the key interactions involve domains C0C2, which demonstrate the greatest effect in activating the thin filament independent of Ca^2+^ (14). Electron microscopy and fluorescence-based single-molecule microscopy assays have suggested a mechanism by which NTDs bind to the thin filament independent of Ca^2+^ concentration and displace tropomyosin (Tm) to expose myosin binding sites, facilitating cross-bridge (XB) formation (17–19). On the other hand, cMyBPC NTD interactions with myosin subfragment 1 (S1, the head or motor domain) and subfragment 2 (S2) have been proposed as distinct mechanisms wherein cMyBPC inhibits XB cycling, independent of NTD thin filament interactions (15, 20). NTD interactions with S1 and S2 effectively tether S1 heads in a sequestered state, decreasing the net pool of force generating S1 heads (20) (Figure 1C). It is now generally accepted that cMyBPC’s regulatory action relies on key residues in the NTDs. The majority of studies have established that NTDs are sufficient to alter cardiac muscle function in vitro, but little is known about the impact of NTDs on cardiac contractile function in vivo.

We and others have shown that the phosphorylation of cMyBPC during neurohormonal stress dynamically enhances in vivo ventricular contractility and accelerates XB kinetics to meet increased circulatory demand (21–25). Similarly, in vitro evidence suggests that C0C2 NTD interactions are also modulated by phosphorylation, with resultant functional effects similar to FL cMyBPC (20). However, it is unknown if C0C2 can affect in vivo contractile function and whether C0C2 would be responsive to adrenergic neurohormonal signaling. Thus, this study investigated the in vivo functional efficacy of C0C2 expression. We hypothesized that the reconstitution of C0C2 NTDs would be as effective as the reconstitution of FL cMyBPC in the neonatal rescue of the HCM phenotype in cMyBPC^–/–^ mice. We present functional evidence that C0C2 reconstitution by gene transfer in cMyBPC^–/–^ mice greatly mitigates the development of the HCM phenotype.

## Results

### Myofilament protein expression and phosphorylation in virus-treated cMyBPC^–/–^ myocardium.

**Figure 2:**
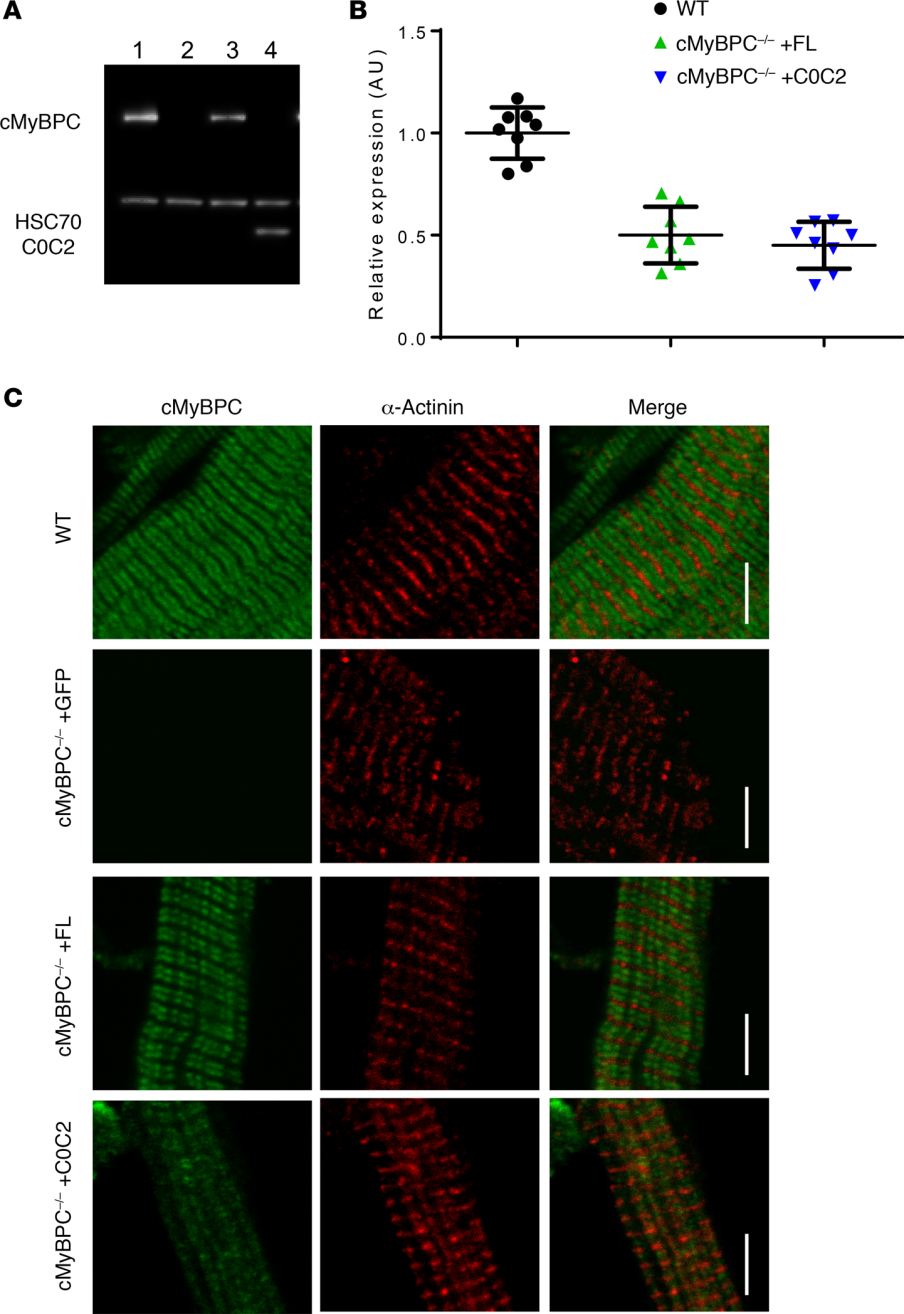
Expression of FL and C0C2 6 weeks after gene transfer. (**A**) Representative Western blot showing the expression of cMyBPC in myofibrils from WT (lane 1), AAV9-GFP–treated cMyBPC^–/–^ (lane 2), AAV9-FL–treated cMyBPC^–/–^ hearts (lane 3), and AAV9-C0C2–treated cMyBPC^–/–^ hearts (lane 4). (**B**) Quantification of cMyBPC and C0C2 expression in WT, AAV9-FL–treated cMyBPC^–/–^ hearts, and AAV9-C0C2–treated cMyBPC^–/–^ hearts. *n* = 8 per group. (**C**) Representative IHC of cMyBPC and α-actinin–stained myofibrils from WT and respective AAV9-treated cMyBPC^–/–^ hearts. Scale bar: 5 μm.

To determine the relative effectiveness of AAV9 gene transfer in cMyBPC^–/–^ mice, cMyBPC expression levels were quantified in AAV9-FL–, AAV9-C0C2–, or AAV9-GFP–treated cMyBPC^–/–^ myocardium at 6 weeks. The relative levels of FL and C0C2 were normalized to age-matched WT samples. The absence of cMyBPC in AAV9-GFP–treated cMyBPC^–/–^ was confirmed by Western blot (Figure 2A). Due to the lack of CTDs, quantification of C0C2 and cMyBPC protein content was performed using whole cell lysates. The average content of cMyBPC in AAV9-FL–treated left ventricular (LV) whole cell lysate samples was 50.1% ± 4.9%, normalized to WT samples (Figure 2B). The average content of C0C2 N-terminal fragment in AAV9-C0C2–treated samples were found to be 45.1% ± 4.1%, normalized to WT (Figure 2B).

The expression and localization of FL and C0C2 in AAV9-FL– and AAV9-C0C2–treated myocardium were further characterized by IHC. It has been well described in mammalian striated muscles that cMyBPC is localized to the A band, decorating the C zones at ~42 nm repeating transverse bands (26, 27). Confocal microscopy does not provide the resolution down to the 42 nm repeats, but it does allow visualization of cMyBPC decoration within the C zone. As expected, AAV9-FL–treated cMyBPC^–/–^ myocardium showed cMyBPC localization at the C zones of each sarcomere, forming characteristic doublet patterns within each sarcomere (Figure 2C). cMyBPC^–/–^ myocardium treated with AAV9-C0C2 resulted in a diffuse staining pattern lacking distinct doublets. This suggests that C0C2 localization is not restricted to the C zone due to the lack of CTDs that anchor to the thick filament.

**Figure 3:**
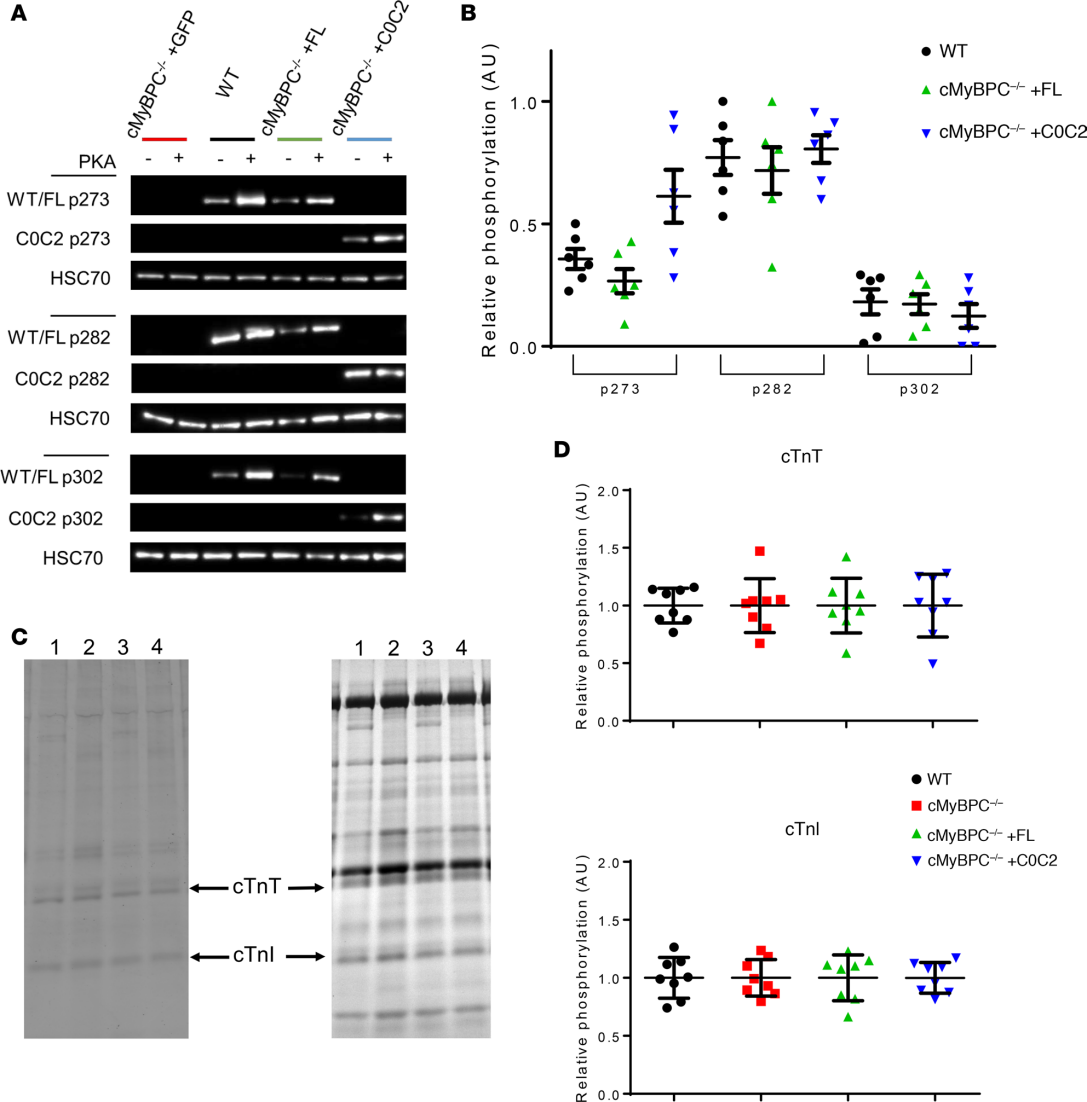
Phosphorylation of myofibril proteins 6 weeks after AAV9-FL and AAV9-C0C2 gene transfer. (**A**) Representative Western blot of cMyBPC phosphorylation at specific phosphoserine residues (p273, p282, p302) from WT and AAV9-treated cMyBPC^–/–^ myofibrils. (**B**) Quantification of relative site-specific cMyBPC phosphorylation, the signal intensities of non–PKA-treated samples were normalized to signal intensities of PKA-treated samples, and PKA-treated phosphorylation level was set as 1 (*n* = 6 per group). (**C**) Representative Pro-Q diamond–stained (left) and Coomassie-stained (right) gel image used to quantify relative phosphorylation and expression of cardiac troponin T (cTnT) and cardiac troponin I (cTnI) from WT (lane 1), AAV9-GFP–treated cMyBPC^–/–^ (lane 2), AAV9-FL–treated cMyBPC^–/–^ hearts (lane 3), and AAV9-C0C2–treated cMyBPC^–/–^ hearts (lane 4). (**D**) Quantification of relative cTnT (top) and cTnI (bottom) phosphorylation level. The Pro-Q signal intensity of each sample was normalized to the total protein Coomassie band intensity, and WT phosphorylation level was set as 1 (*n* = 8 per group).

The phosphorylation of cMyBPC in response to adrenergic stimulation is a vital response during periods of increased cardiac demand (23, 24). The relative levels of cMyBPC phosphorylation at 3 key phosphorylation sites in the M domain (mouse residues p273, p282, and p302) in FL and C0C2 were characterized to determine whether AAV9-treated cMyBPC^–/–^ myocardium has normalized basal cMyBPC phosphorylation levels. AAV9-FL– and AAV9-C0C2–treated cMyBPC^–/–^ hearts showed similar levels of phosphorylation in p282 and p302 when compared with WT hearts (Figure 3, A and B). AAV9-C0C2–treated cMyBPC^–/–^ hearts showed p273 phosphorylation levels that appeared elevated compared with WT hearts, but this was not statistically significant. Overall, this shows that the FL and C0C2 proteins expressed in cMyBPC^–/–^ hearts are competent protein kinase A (PKA) substrates. Analysis of the relative phosphorylation and abundance of cardiac troponin T (cTnT) and cardiac troponin I (cTnI) in WT, AAV9-GFP–treated cMyBPC^–/–^, and AAV9-FL– and C0C2-treated cMyBPC^–/–^ myocardium found no significant differences between these groups (Figure 3, C and D).

### Effect of FL and C0C2 gene transfer on cardiac morphology and in vivo function.

**Figure 4:**
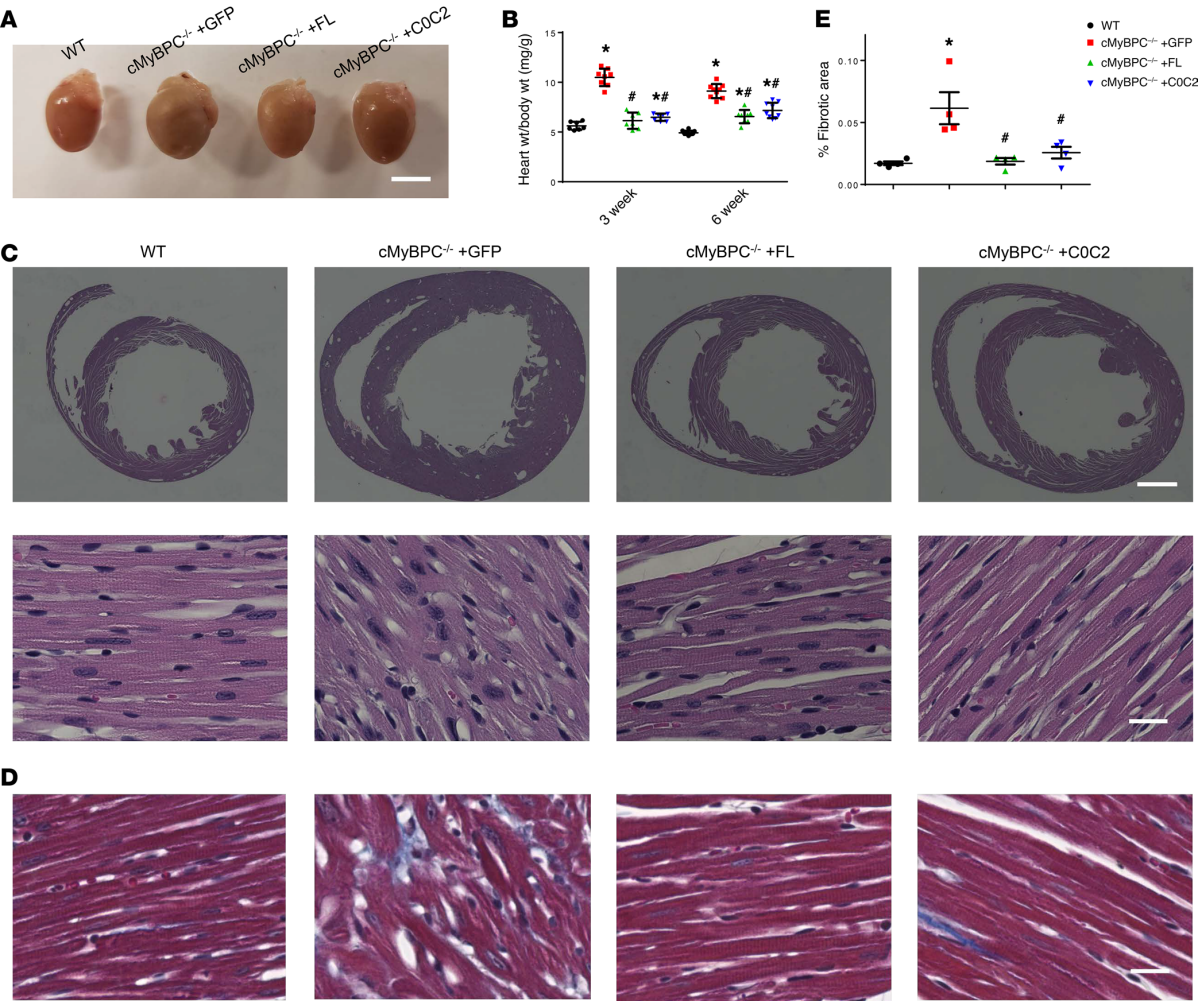
Histologic and gross characterization of 6-week AAV9-treated hearts. (**A**) Representative hearts from WT and AAV9-treated cMyBPC^–/–^ mice. Scale bar: 5 mm. (**B**) Quantification of heart (wet weight) to body weight ratio of WT and AAV9-treated cMyBPC^–/–^ groups (*n* = 7–10). (**C**) Representative histologic cross sections of WT and AAV9-treated cMyBPC^–/–^ hearts from the mid-LV and stained with H&E. Scales bars: 1 mm (top) and 20 μm (bottom). (**D**) As above, stained with Masson’s trichrome. Scale bar: 20 μm. (**E**) Quantification of fibrosis from Masson’s trichrome cardiac sections, analyzed from 4 mice per group, calculated from 8 areas of interest within a midventricular horizontal section (2 areas each from anterior, lateral, and posterior free walls and septum). Values are expressed as mean ± SEM. Significance was determined by 1-way ANOVA with Tukey’s multiple comparisons test. *P < 0.05 versus WT group; ^#^*P* < 0.05 versus cMyBPC^–/–^ + GFP.

Cardiac morphology was examined to determine the in vivo phenotypic effects of FL cMyBPC and C0C2 gene transfer. Without intervention, cMyBPC^–/–^ mice are born with phenotypically normal hearts but develop cardiac hypertrophy days after birth. Consistent with previous findings (28), AAV9-GFP–treated cMyBPC^–/–^ hearts displayed severe cardiomegaly at 3 and 6 weeks, whereas AAV9-FL– and AAV9-C0C2–treated cMyBPC^–/–^ hearts appeared to be similar in size to WT hearts (Figure 4A). Vehicle-treated cMyBPC^–/–^ mice were found to have significantly higher heart weight/body weight ratios (HW:BW) at 3 (10.49 ± 0.31 mg/g) and 6 (9.12 ± 0.24 mg/g) weeks old when compared with age-matched 3-week-old (5.62 ± 0.16 mg/g, *P* < 0.05) and 6-week-old (4.92 ± 0.07 mg/g, *P* < 0.05) WT mice. The HW:BW of AAV9-FL–treated cMyBPC^–/–^ mice were significantly reduced to 6.14 ± 0.31 mg/g and 6.55 ± 0.24 mg/g at 3 and 6 weeks, respectively. Furthermore, the AAV9-FL–treated cMyBPC^–/–^ HW:BW ratio was normalized to WT levels at the 3-week time point. The HW:BW of AAV9-C0C2–treated cMyBPC^–/–^ mice was also significantly reduced to 6.49 ± 0.14 mg/g and 7.16 ± 0.26 mg/g at 3 and 6 weeks, respectively (Figure 4B).

In agreement with previous findings, histological examination showed extensive signs of hypertrophic pathology in vehicle-treated cMyBPC^–/–^ hearts, including myocyte disarray and increased interstitial fibrosis, features that were not observed in the AAV9-FL– and AAV9-C0C2–treated cMyBPC^–/–^ hearts (Figure 4C). Cardiac fibrosis, assessed by Masson’s trichrome staining, was significantly higher in AAV9-GFP–treated cMyBPC^–/–^ hearts (0.065% ± 0.016% versus WT 0.017% ± 0.001% *P* < 0.05), which were significantly reduced in AAV9-FL–treated (0.019% ± 0.003%, *P* < 0.05) and AAV9-C0C2–treated (0.026% ± 0.005% *P* < 0.05) cMyBPC^–/–^ hearts (Figure 4, D and E). Echocardiographic analysis confirmed the reduction of pathologic hypertrophy in the AAV9-FL– and AAV9-C0C2–treated cMyBPC^–/–^ mice, as demonstrated by the significant reduction of LV wall thicknesses and chamber dimensions when compared with AAV9-GFP–treated cMyBPC^–/–^ mice (Table 1 and Table 2). However, AAV9-C0C2–treated cMyBPC did show signs of hypertrophy, as evidenced by significantly increased anterior wall thickness in systole (AWTs) and end systolic diameter (ESD) at the 6-week time point, when compared with WT (Table 2).

**Figure 5:**
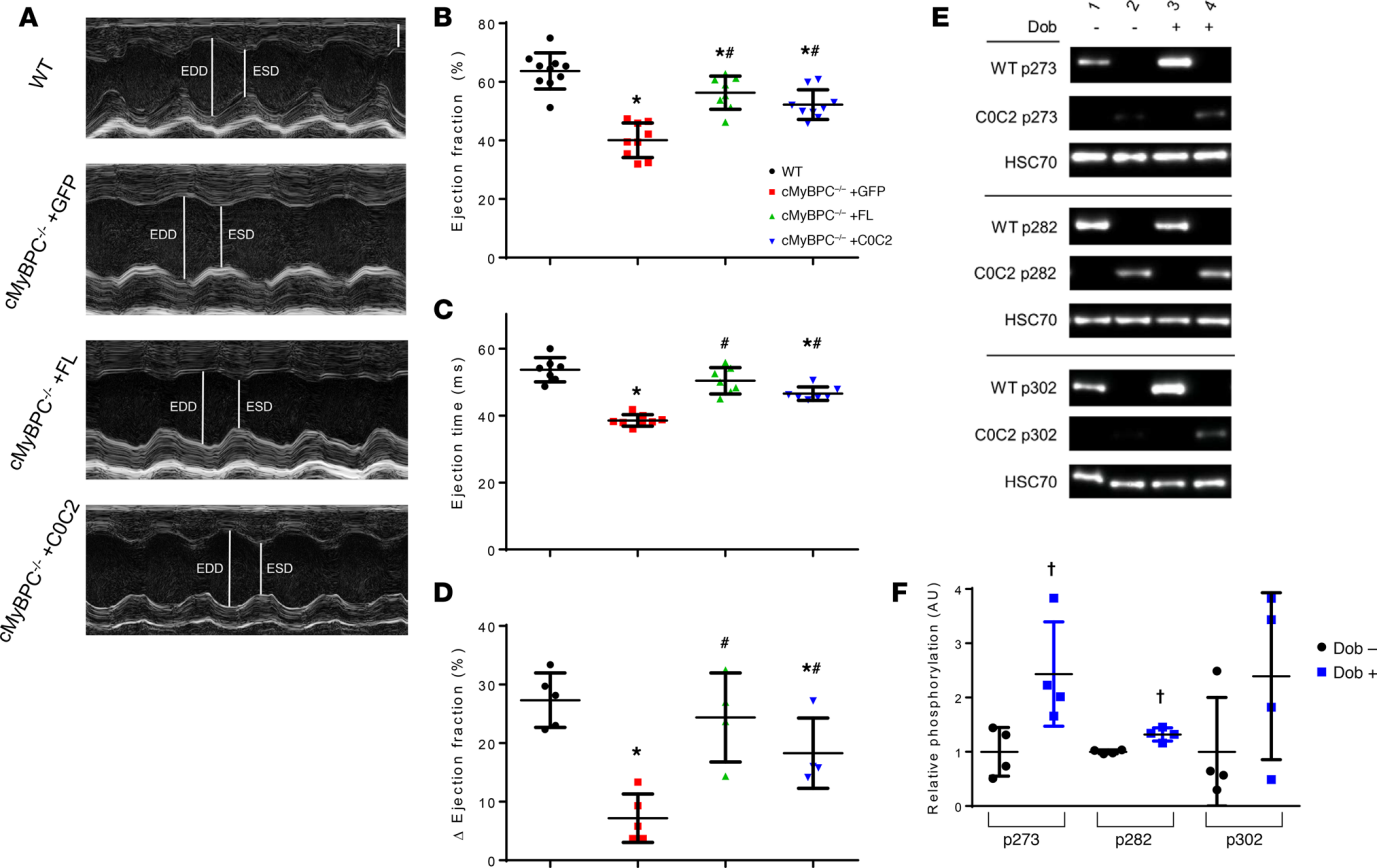
Effects of AAV9-FL and –C0C2 gene transfer on cMyBPC^–/–^ in vivo cardiac function at 6 weeks. (**A**) Representative 2D echocardiography images acquired in M-mode along the parasternal short-axis view. Scale bar: 1 mm. (**B** and **C**) Quantification of basal systolic ejection fraction (**B**) and ejection times (**C**) of WT and AAV9-treated cMyBPC^–/–^ mice from 8–11 animals per group. (**D**) Quantification of net increase in ejection fraction after 10 mg/kg i.p. bolus dobutamine injection to assess acute β-adrenergic stress response from 4–5 animals per group. Values are expressed as mean ± SEM. Significance was determined by 1-way ANOVA with Tukey’s multiple comparisons test. *P < 0.05 versus WT group; ^#^*P* < 0.05 versus cMyBPC^–/–^ +GFP. (**E**) Representative Western blot showing S273, S282, and S302 phosphorylation of control (Dob–) and dobutamine infused (Dob+) WT and AAV9-C0C2–treated cMyBPC^–/–^ hearts. (**F**) Quantification of relative phosphorylation of AAV9-C0C2–treated control (Dob–) and dobutamine infused (Dob+) hearts at phosphoserines 273, 282, and 302. *n* = 4 per group. †*P* < 0.05 by unpaired *t* test analysis.

The effect of AAV9-FL and AAV9-C0C2 gene transfer on in vivo contractile function was also assessed by echocardiography. Consistent with previous studies, vehicle-treated cMyBPC^–/–^ mice showed significantly impaired systolic function, as measured by reductions in ejection fraction (EF) and fractional shortening (FS) at both time points when compared with WT mice. In contrast, the systolic function of AAV9-FL–treated cMyBPC^–/–^ mice was improved, resulting in significantly higher EF and FS at both time points (Table 1 and Table 2). In the AAV9-C0C2–treated mice, there was a significant recovery of EF and FS at the 6-week time point compared with vehicle-treated cMyBPC^–/–^ mice (Figure 5, A and B). The absence of cMyBPC is also thought to shorten XB lifetime and reduce myofilament rigidity, contributing to reduced systolic tension generation (29). Accordingly, ejection time (ET) was significantly abbreviated in the vehicle-treated cMyBPC^–/–^ mice, contributing to the reduction in cardiac output. In the AAV9-C0C2 and -FL cMyBPC^–/–^ mice, ET was significantly longer (50.49 ± 1.76 ms and 46.74 ± 1.31 ms, respectively) compared with vehicle-treated cMyBPC^–/–^ mice (38.70 ± 0.47 ms, *P* < 0.05) and normalized to near WT levels (Figure 5C).

Previous work showed that PKA-mediated acceleration of XB kinetics was absent in cMyBPC^–/–^ myocardium and that this effect is largely mediated by phosphorylation of key serine residues within the M domain (Figure 1A). Thus, we investigated whether expressing FL and C0C2 in cMyBPC^–/–^ could augment the limited contractile reserve found in cMyBPC^–/–^ mice. As shown above, the C0C2 protein contains the critical serine phosphorylation sites within the M domain and is competent as a PKA substrate (Figure 3B), suggesting that the expression of C0C2 in cMyBPC^–/–^ hearts could also rescue the diminished contractile reserve of cMyBPC^–/–^ mice. As expected, we found vehicle-treated cMyBPC^–/–^ mice exhibited significantly reduced contractile reserve in response to dobutamine infusion (Figure 5D). In contrast, dobutamine infusion increased WT EF by 27.3% ± 2.1%, which was nearly matched by the AAV9-FL–treated cMyBPC^–/–^ mice (24.4% ± 3.8%, *P* < 0.05 versus AAV9-GFP–treated cMyBPC^–/–^). Notably, AAV9-C0C2–treated cMyBPC^–/–^ mice also showed significantly greater contractile enhancement compared with AAV9-GFP–treated cMyBPC^–/–^ (18.3% ± 3.0% versus 7.2% ± 1.8%, respectively; *P* < 0.05). Comparison of site-specific phosphorylation at p273, p282, and p302 residues in unpaired control and dobutamine-treated hearts demonstrated increased levels phosphorylation at p273 and p282 after dobutamine infusion (Figure 5, E and F). Though p302 changes did not reach statistical significance, this is likely due to variability in basal phosphorylation levels.

**Figure 6:**
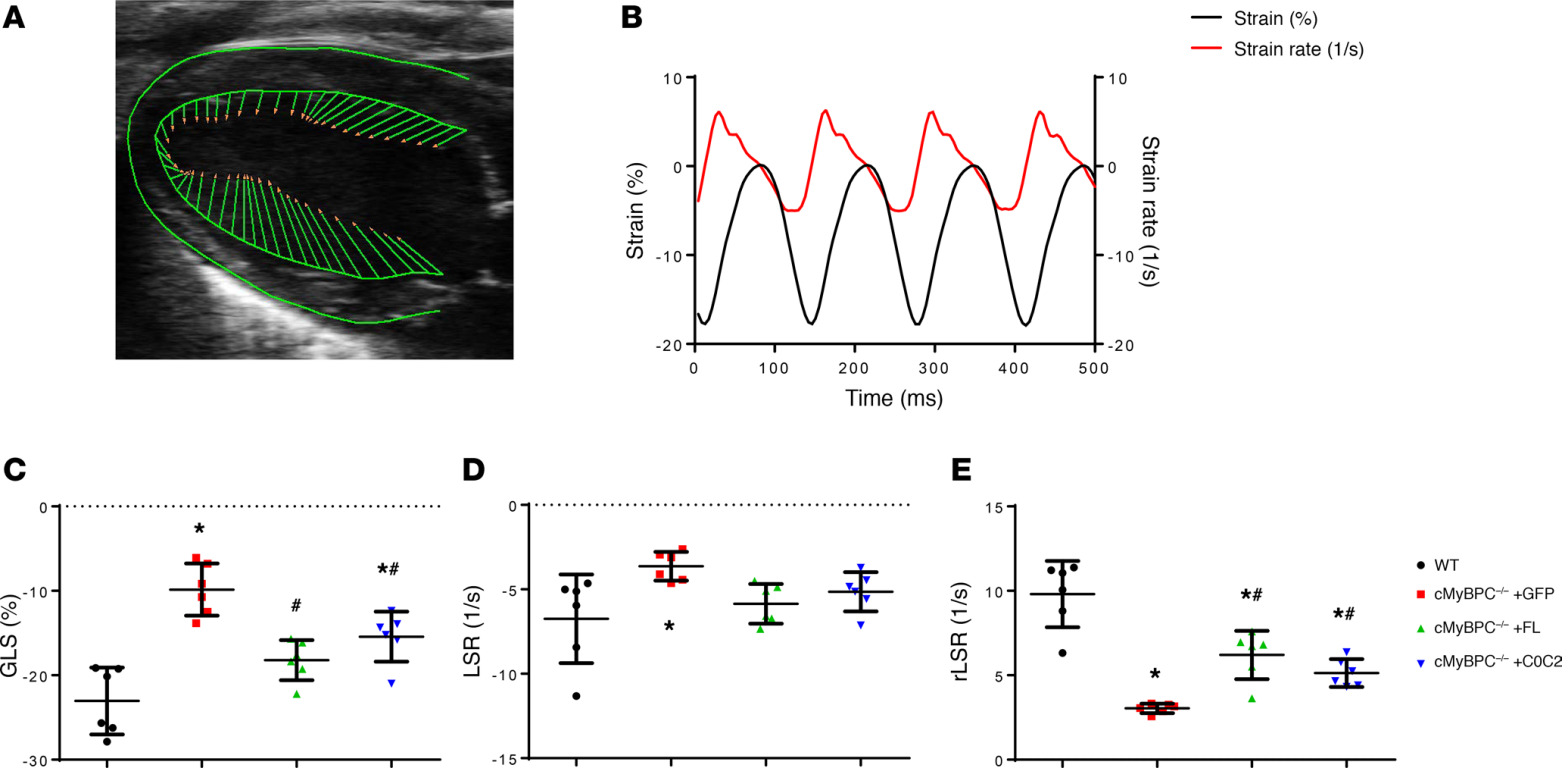
Speckle-tracking strain analysis of 6-week AAV9-treated mice. (**A**) Representative B-mode image in parasternal long-axis view in which myocardium is traced and vector arrows of longitudinal strain along the endocardial border is displayed. (**B**) Representative average longitudinal strain (black) and strain rate (red) along the left ventricular myocardium. (**C–E**) Quantification of global longitudinal strain (GLS) (**C**), peak longitudinal strain rate (LSR) (**D**), and peak reverse longitudinal strain rate (rLSR) (**E**) in WT and AAV9-treated cMyBPC^–/–^ mice, from 6 animals per group. Negative values indicate contraction; positive values indicate relaxation. Values are expressed as mean ± SEM. Significance was determined by 1-way ANOVA with Tukey’s multiple comparisons test. *P < 0.05 versus WT group; ^#^*P* < 0.05 versus cMyBPC^–/–^ + GFP.

To further study the effects of AAV9-FL and AAV9-C0C2 on cMyBPC^–/–^ systolic and diastolic function, we assessed speckle-tracking echocardiography–based (STE-based) global longitudinal strain (GLS), longitudinal strain rate (LSR), and reverse longitudinal strain rate (rLSR) (Figure 6, A and B). Myocardial strain analysis has been well established as a rapid noninvasive technique for assessing intrinsic myocardial contractility in humans and rodents (30, 31). We found GLS to be significantly higher in AAV9-FL– and AAV9-C0C2–treated cMyBPC^–/–^ mice, compared with AAV9-GFP cMyBPC^–/–^ (Figure 6C). These results correlate well with our findings by conventional echocardiography: FL cMyBPC and C0C2 reconstitution are of benefit in improving the HCM phenotype. Likewise, rLSR significantly increased after FL and C0C2 reconstitution, indicating partial recovery of diastolic function (Figure 6E).

### Effect of FL and C0C2 cMyBPC reconstitution on in vitro myocardial mechanics.

**Figure 7:**
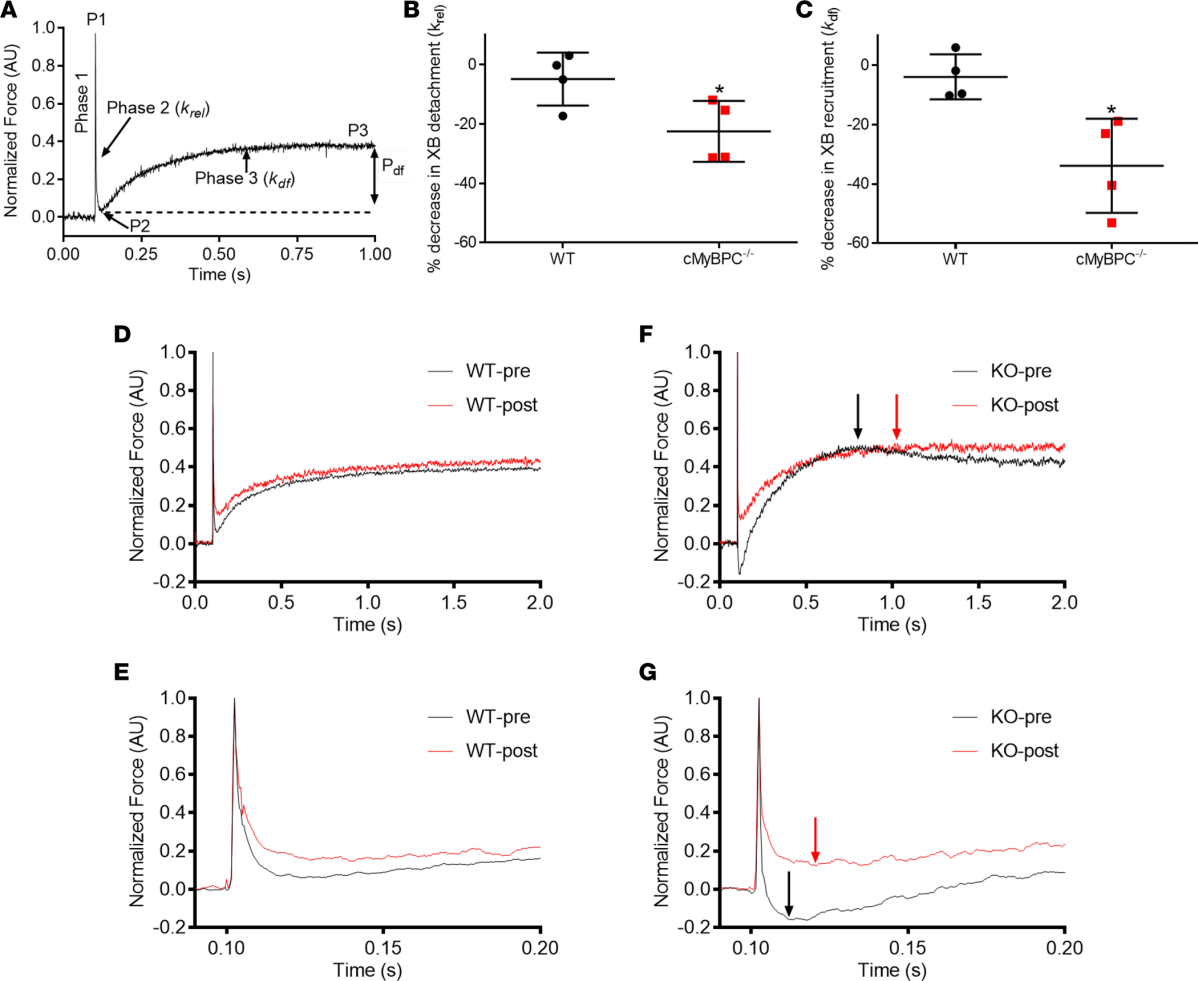
Stretch activation responses in WT and cMyBPC^–/–^ myocardial preparations before and following C0C2 peptide incubation. (**A**) Shown is a typical force trace in response to a sudden 2% stretch in muscle length (ML) in an isometrically contracting WT cardiac muscle preparation at a SL of 2.1 μm. Highlighted are the important phases of the force response and various stretch activation parameters that are derived from the response. Phase 1 shows the immediate increase in force in response to the sudden 2% stretch in ML. P1 is the magnitude of the immediate force response that is measured from the prestretch isometric steady-state force to the peak of phase 1. Phase 2 represents the rapid decline in the force with a dynamic rate constant *k*_rel_, an index of XB detachment rate. Phase 3 represents the delayed force development with a dynamic rate constant *k*_df_, an index of XB recruitment rate (please see Methods for further details). Measurements in the skinned ventricular preparations were first made under basal conditions (no peptide incubation). Measurements were again made on the same preparations following a 10-minute incubation with 1.0 μM C0C2 peptide incubation. (**B** and **C**) Percent decreases in the rates of XB detachment (*k*_rel_) and XB recruitment (*k*_df_) following a 10-minute incubation with 1.0 μM C0C2 peptide incubation. *n* = 4 hearts per group. Incubation with C0C2 peptide resulted in significant slowing of *k*_rel_ and *k*_df_ in cMyBPC^–/–^ but not in the WT preparations. (**D** and **F**) Representative WT (**D**) and in cMyBPC^–/–^ (**F**) stretch activation traces highlighting the changes in *k*_df_, which was significantly slowed in cMyBPC^–/–^ group, as indicated by a delay in the achievement of peak of XB recruitment phase (red arrow) for trace obtained after C0C2 incubation versus black arrow for trace obtained before C0C2 incubation. No such delay in the achievement of peak of XB recruitment phase was observed in the WT group. (**E** and **G**) Expanded views are shown below to demonstrate that *k*_rel_ is significantly slowed following C0C2 peptide incubation in the cMyBPC^–/–^ group but not in the WT group, as indicated by delayed achievement of the nadir of the force decay in cMyBPC^–/–^ preparations following C0C2 incubation. Values are expressed as mean ± SEM. Significance was determined by 1-way ANOVA with Tukey’s multiple comparisons test. *P < 0.05 by paired *t* test comparing *k*_rel_ and *k*_df_ rates before and after incubation with C0C2 peptide.

Skinned myocardium was isolated from hearts of each mouse line to determine steady-state force generation and dynamic XB kinetics. The lack of cMyBPC in the sarcomere has been shown to significantly accelerate baseline XB kinetics (32). We previously demonstrated that gene transfer and exogenous cMyBPC protein reconstitution in adult cMyBPC^–/–^ mice resulted in the rescue of myofilament XB dysfunction (9). Here, we investigated the effects of gene transfer by reconstitution of FL and C0C2 cMyBPC in neonatal mice. Consistent with our previous studies, skinned myocardial preparations from AAV9-GFP–treated cMyBPC^–/–^ hearts showed significantly accelerated rates of stretch-induced XB detachment (*k*_rel_) and XB recruitment (*k*_df_) (Figure 7 and Table 3). In contrast, preparations from AAV9-FL–treated cMyBPC^–/–^ hearts displayed significantly slower XB kinetics and displayed similar stretch activation properties compared with WT preparations, which correlated well with our previous rescue of adult cMyBPC^–/–^ using cMyBPC reconstitution (9). Interestingly, AAV9-C0C2–treated cMyBPC^–/–^ preparations also displayed significantly slower XB kinetics compared with AAV9-GFP–treated cMyBPC^–/–^, as measured by *k*_rel_ and *k*_df_ (Figure 7 and Table 3). No significant differences in maximal Ca^2+^ activated force (F_max_), Ca^2+^ sensitivity (pCa [pCa = –log(Ca^2+^)_free_] required for the generation of half-maximal force generation; pCa_50_), or cooperativity (Hill coefficient of the force-pCa relationship; *n*_H_) of force production were observed between the groups (Table 4 and Supplemental Figure 1; supplemental material available online with this article; https://doi.org/10.1172/jci.insight.130182DS1).

Due to the lack of anchoring domains in C0C2 protein, there may be some loss of free-floating C0C2 during the skinning procedure for AAV9-treated samples, thereby reducing the apparent effect on myofilaments. To account for this and to characterize the effect of the C0C2 protein on in vitro XB kinetics in the presence of endogenous cMyBPC, we incubated WT and cMyBPC^–/–^ skinned preparations with 1 μM exogenous recombinant C0C2 protein. In the intact myocyte, endogenous cMyBPC concentration is estimated to be in the low micromolar range (13). Because we found AAV9-driven C0C2 expression did not exceed levels of native cMyBPC found in WT mice, we examined exogenous recombinant C0C2 protein effects at comparable and physiologically relevant concentrations. After C0C2 incubation, F_max_, pCa_50_, and *n*_H_ were unchanged compared with respective preincubation WT and cMyBPC^–/–^ groups. We did observe a small but statistically significant increase in F_min_ in cMyBPC^–/–^ (4.91% ± 1.66% of F_max_) (Supplemental Table 1). We also examined the effects of exogenous C0C2 on in vitro XB kinetics using stretch activation experiments. Exogenous C0C2 concentration was selected based on previous in vitro motility and binding studies showing that 1 μM C0C2 sensitizes WT myofilament to Ca^2+^ at short sarcomere length (SL) (33) and inhibits actin-sliding velocity (34). Remarkably, after incubating cMyBPC^–/–^ skinned preparations, *k*_rel_ decreased by 22.6% (*P* < 0.05) and *k*_df_ decreased by 35% (*P* < 0.05) at matched submaximal levels of Ca^2+^ activation (pCa 6.1) (Supplemental Table 2). No significant XB kinetic changes were observed in corresponding paired WT groups before and after C0C2 incubation.

## Discussion

Autosomal-dominant mutations in *MYBPC3* are the most common causes of inheritable HCM (3). Many *MYBPC3* mutations are truncations that result in reduced cMyBPC expression in the myocardium, which leads to disrupted actin-myosin interactions and hypercontractility (6, 35). Thus, there is a compelling rationale for restoring cMyBPC regulatory function as the most direct way to normalize contractility in cases where a cMyBPC deficiency is detected. Previous in vitro studies have identified the NTDs of cMyBPC as the regions involved in its interactions with actin and myosin (33, 36, 37). Our current study investigated whether gene transfer of the C0C2 NTD fragment could mitigate the HCM phenotype in cMyBPC^–/–^ mice by normalizing in vivo contractile function in a manner similar to that of FL cMyBPC.

This study is the first to our knowledge to characterize the effects of the C0C2 domains of cMyBPC in vivo using a cMyBPC^–/–^ mouse model of HCM. Previously, we showed that the reconstitution of FL cMyBPC in adult cMyBPC^–/–^ mice can significantly improve contractile function and reduce ventricular hypertrophy. As expected, FL cMyBPC reconstitution by AAV9 gene transfer in cMyBPC^–/–^ neonates significantly reduced the severity and delayed the onset of the HCM phenotype. Importantly, our results show C0C2 to be sufficient in ameliorating the HCM phenotype. We found C0C2 expression in cMyBPC^–/–^ mice significantly altered contractile function at the whole organ and myofilament levels, which greatly reduced LV chamber dimensions and wall thicknesses when compared with AAV9-GFP–treated cMyBPC^–/–^ hearts (Table 1 and Table 2). The reduction of hypertrophy was accompanied by significant reductions of the HW:BW ratio, enhanced systolic function (EF and FS), and the absence of overt histological signs of HCM (i.e., myocardial disarray and fibrosis). The functional enhancements in C0C2- and FL-treated cMyBPC^–/–^ are likely due, in part, to a recovery of ET. Previous studies have shown that cMyBPC interactions with myosin contribute to XB stability and prolong the XB duty cycle (29, 38). Mechanistically, our data from in vitro skinned fiber experiments support the in vivo findings. Fiber preparations from AAV9-FL– and AAV9-C0C2–treated cMyBPC^–/–^ hearts showed significantly slower rates of XB detachment (*k*_rel_) compared with preparations from AAV-GFP–treated cMyBPC^–/–^ hearts. The accelerated *k*_rel_ is likely due to the absence of cMyBPC that would otherwise provide mechanical rigidity to sarcomere lattices, prolonging XB lifetimes. NTD fragments are also known to bind myosin and slow ATPase activity (34), which may result in the slower XB turnover and prolonged ET we observed in AAV9-C0C2–treated cMyBPC^–/–^ hearts. Another possibility that explains the prolonged ET could be NTD interactions with actin, which can displace Tm into its open state (18). Increased actin binding by NTDs could extend the time that Tm is in the open states, since Ca^2+^ in the myocyte falls in early diastole, delaying the cooperative thin filament deactivation, and prolonging ejection, as suggested by van Dijk et al. (39). We also observed significant increases in GLS in the rescue groups. These parameters are well correlated with the extent of hypertrophy and decreases in intrinsic myocardial contractility, which further validates the effectiveness of C0C2 in reversing systolic dysfunction. We also found the ratio of mitral peak velocity of early to late filling (E/A ratio) to be normalized after AAV9-FL and -C0C2 gene transfer, suggesting there is some improvement in cMyBPC^–/–^ diastolic dysfunction. Furthermore, FL and C0C2 gene transfer resulted in partial recovery of rLSR in the STE analysis, which describes the peak myocardial LSR during early diastolic filling. This parameter has been shown to be a sensitive measure of relaxation that can better detect “subclinical” diastolic dysfunction (40). When compared with AAV9-GFP–treated cMyBPC^–/–^ mice, the significant increases in rLSR in the AAV9-FL– and AAV9-C0C2–treated cMyBPC^–/–^ mice suggest that C0C2-mediated myofilament modulation may improve diastolic ventricular refill in the HCM phenotype driven by cMyBPC^–/–^.

It is also possible that the changes in cardiac function in AAV9-C0C2–treated cMyBPC are due to mechanisms distinct from that of FL, since NTDs can exert effects outside of the C zone and high concentrations of NTDs (>10 μM) have been shown to significantly increase Ca^2+^-independent thin filament activation (13, 14, 33). Here, we found that the skinned fibers from AAV9-C0C2–treated cMyBPC^–/–^ hearts did not show altered isometric steady-state Ca^2+^-activated maximal force generation or changes in Ca^2+^ sensitivity (Table 4). We also confirmed our findings in fiber experiments incubated with exogenous recombinant C0C2, where XB kinetics were significantly slower after incubation (Supplemental Table 1 and Supplemental Table 2). The discrepancy described between the results here and previous examination of NTD on cMyBPC^–/–^ fibers are likely due to differences in NTD concentration. Our findings suggest in vivo C0C2 expression did not reach high enough concentrations to significantly activate the thin filament independent of Ca^2+^, which would lock sarcomeres in a force-generating state, preventing proper relaxation and impairing diastolic filling. Thus, the enhanced in vivo contractile function is most likely a result of a return to normalized XB kinetics, XB duty ratio, and timing of cooperative thin filament deactivation, as opposed to changes in steady-state force production.

cMyBPC phosphorylation is a critical modulator of in vivo contractile acceleration in response to β-adrenergic stimulation (23, 41). We found that AAV-C0C2–treated cMyBPC^–/–^ showed significantly recovered contractile reserve in response to dobutamine, as evidenced by Δ EF (Figure 5D). We hypothesize that this recovery is likely due to the phosphorylation of S273, S282, and S302, which are the most well studied cMyBPC β-adrenergic–mediated PKA phosphorylation sites (23, 42–44). The ability of C0C2 to improve contractile reserve is an important property because diminished contractile reserve is thought to be a significant contributor to HF pathogenesis (23, 41) and has been shown to be a poor prognostic indicator in human HF and cardiomyopathies (45). It is promising that these regulatory residues retain their functional response to upstream signaling in vivo and that AAV9-treated hearts have p273, p282, and p302 phosphorylation levels similar to those of WT hearts (Figure 3B). Studies have shown phosphorylation at these residues is cardioprotective (44, 46–48), and their phosphorylation levels are decreased in human and experimental HF (49–51). Thus, our results support the notion that manipulation of cMyBPC N-terminal phosphorylation has therapeutic potential for the treatment of HF and that this potential may extend to other truncated variants of cMyBPC that retain the M domain.

As mentioned in the introduction, native cMyBPC is anchored to thick filaments in a strict stoichiometric pattern within the C zone, resulting in a relatively low cMyBPC to myosin ratio (~1:8) and cMyBPC to actin ratio (~1:12) (15, 52). The low ratio of cMyBPC to actin is thought to optimize the level of thin filament activation while limiting the competition for XB binding sites with myosin. We and others have shown that cMyBPC cannot be overexpressed beyond native levels due to full occupancy of anchoring sites within the C zone (9, 53). A potential advantage of expressing only the NTDs is to eliminate this restriction, allowing C0C2 to interact with myosin heads and segments of the thin filament normally out of reach of anchored endogenous cMyBPC. Clinically, this feature would be best used in patients with cMyBPC missense mutations, which are found in the majority of childhood-onset HCM (54). The early-onset nature of these missense mutations implies a greater degree of mutation pathogenicity, relative to the truncation mutations predominantly associated with adult-onset HCM. Importantly, missense mutations produce the FL cMyBPC but result in the substitution of one amino acid for another. This would result in a heterogeneous population of WT and mutant cMyBPC decorating the C zone. By using NTDs, transgene products could deliver contractile modulation without having to compete with mutant cMyBPC. It will therefore be of clinical interest to determine the precise mechanisms by which NTDs modulate contractile function so as to design NTD-based treatments to counter specific HCM mutations.

Although this study suggests that FL and C0C2 gene transfer was effective in delaying and significantly mitigating pathologic HCM phenotypes, the following limitations should be considered. The dose of AAV9 chosen for this study was designed to directly compare the effects of FL and C0C2 at equivalent vector dosing and protein expression levels. AAV9 vectors are generally considered to have high safety and tolerability in animal studies and human therapies (10, 55, 56). Based on previous studies, we selected our dose with respect to our viral titer with additional consideration to injection volume. Unfortunately, high-volume (>40 μL) injections resulted in unacceptably high mortality rates. As a result, we did not achieve expression levels of reconstituted protein that were matched to that of WT cMyBPC expression. For this reason, our FL and C0C2 gene transfers did not result in complete rescue of cMyBPC^–/–^ phenotype and restoration of function to WT levels. This is consistent with results of previous studies showing a that threshold cMyBPC expression is needed to maintain normal function without deleterious haploinsufficiency (9, 10, 57). Thus, it is likely our AAV9 gene transfers did not completely rescue the cMyBPC^–/–^ mice due to inadequate levels of FL and C0C2 expression. Additionally, it will be important in future studies to examine the effect of C0C2 gene transfer on adult cMyBPC^–/–^ mice to determine if C0C2 can rescue the HCM phenotype and induce reverse remodeling.

In summary, the idea that NTD fragments can mimic the effects of FL cMyBPC is supported by numerous studies that have characterized NTD functional effects in vitro. For the first time to our knowledge, we show that the C0C2 NTD can exert these effects in vivo and is capable of dynamically regulating contractile function to meet increased cardiac demand. This is evidenced by the fact that AAV9-C0C2 significantly mitigated and delayed development of the HCM phenotype in the cMyBPC^–/–^ mouse model. The above results strongly support the idea that C0C2 domains are sufficient for modulating sarcomeric contractility in a manner similar to that of FL cMyBPC and, therefore, may be useful in treating human cases with reduced cMyBPC expression.

## Methods

### Transgenic animals.

cMyBPC^–/–^ (28) mice were generated previously and are of the same 129SV background as WT mice used. cMyBPC^–/–^ mice rapidly develop a severe, early-onset HCM-related phenotype that remains relatively stable over time. cMyBPC^–/–^ mice survive well into adulthood (>1 year) and are fertile (28).

### AAV9 production and administration.

AAV9 pseudotyped vectors expressing EGFP, FL cMyBPC, and C0C2 cMyBPC were designed under the control of a cTnT promoter (pENN.AAV.cTNT.PI.eGFP.WPRE.rBG). Vectors were produced by the Penn Vector Core at the University of Pennsylvania (Philadelphia, Pennsylvania, USA), as described previously (58). The specific murine FL and C0C2 sequences used in this study are listed in Supplemental Figure 2. During the first 36 hours following birth, neonatal pups were anesthetized by hypothermia and received i.v. administration of 2 × 10^11^ genome copy (GC)/mouse via the temporal facial vein using a 33G needle under standard Biosafety Level 1 (BSL-1) and Animal Biosafety Level 1 (ABSL-1) conditions.

### In vivo cardiac function.

Noninvasive transthoracic echocardiography was performed by an experienced research technician blinded to the study groups on anesthetized (1%–2% isoflurane) 3-week- and 6-week-old mice as previously described (59) using a Vevo 3100 ultrasonography 40 MHz transducer (Visual Sonics) to acquire 2D B mode, M mode, and Doppler images of the left ventricle. Images were analyzed using Vevo Lab 3.1.1 (Visual Sonics). The LV parameters EF, FS, anterior and posterior wall thickness, diameter, and LV mass were measured over 4–5 cardiac cycles from midventricle M-mode images, using the leading edge method. End-systolic dimensions were measured at the peak of the posterior wall motion; end-diastolic dimensions were measured at the time point corresponding to the start of the QRS complex on simultaneous ECG recordings. ET and MV peak velocities E (mitral peak velocity of early filling) and A (mitral peak velocity of late filling) were measured from apical 4-chamber PW Doppler images. STE-based longitudinal strain parameters (Figure 6, A–E) were measured from parasternal long-axis B mode video using Vevo Lab’s built-in semiautomated Vevo Strain analysis.

### Histological analysis of cardiac tissue.

For histological analysis, hearts were perfused with 4% paraformaldehyde (PFA) in anesthetized (4% isoflurane) 6-week-old animals from each group. Hearts were excised and further fixed in a solution of 4% PFA for an additional 48–72 hours at room temperature (RT) before embedding in paraffin for 5-μm serial sectioning and staining as previously described (35). Cross sections were stained with H&E and Masson’s trichrome. Quantitative analysis of trichrome-stained sections was performed using ImageJ (NIH) macro by Kennedy el al. that measured pixels where blue intensity exceeded red intensity by > 120% (60, 61). For each heart, the percent area of fibrosis was obtained from 8 total areas of interest from a midventricular section, with 2 areas of each from the anterior, posterior, and lateral free walls and septum. The total percent area of fibrotic area out of total area examined was averaged from 4 hearts for each mouse line.

### Western blot and Pro-Q analysis of myocardial samples.

Myocardial proteins were studied as previously described (59). Briefly, hearts were excised from anesthetized (3%–4% isoflurane) mice, flash frozen in liquid N_2_, and stored at –80°C for later quantification of protein expression and phosphorylation. Frozen ventricular tissues were thawed in homogenization buffer containing protease and phosphatase inhibitors (PhosSTOP and cOmpleteULTRA Tablets, MilliporeSigma) at 4°C; they were then homogenized. Myofibril and whole cell fraction protein samples were reduced and heated to 90°C for 5 minutes. Sample protein concentrations were determined by BCA protein colorimetric assay (Pierce BCA Protein Assay Kit, Thermo Fisher Scientific) and diluted to 1 μg/μL. A total of 5 μL of solubilized myofibrils was loaded and electrophoretically separated using TruPAGE precast 4%–12% gels (MilliporeSigma) at 180 V for 45 minutes. Proteins were transferred to PVDF membranes for Western blot analysis (Santa Cruz Biotechnology Inc.; cMyBPC, 1:1000, catalog sc-137237; HSC70, 1:2000, catalog sc-7298; custom antibodies by 21st Century Biochemicals, polyclonal rabbit IgG generated against cMyBPC phosphorylated S273 (1:10,000), S282 (1:10,000), and S302 (1:2000) using antigen peptides C-Ahx-AFRRT[pS]LAGAGRR-amide, C-Ahx-GAGRR{T/I}[pS]DSHED{A/T}G-amide, and C-Ahx-SLLKKRD[pS]FRRDSKL-amide, respectively. HSC70 was used as loading control for all Western blots. Control experiments demonstrating stable levels of HSC70 expression in samples from cMyBPC^–/–^ and AAV9-treated hearts was performed by comparison with GAPDH expression (ABclonal, GAPDH, 1:1000 catalog AC002) (Supplemental Figure 4). Samples from 6 mice per group were used in PKA experiments. PKA samples were incubated at 30°C for 1 hour, in a solution containing the catalytic subunit of bovine PKA at a concentration of 0.15 U/μg myofibrils (PKA^+^) before being reduced and heated to 90°C for 5 minutes, as previously described (41, 62). Control samples were incubated in the same conditions in the absence of PKA (PKA^–^). PKA^–^ sample Western blot p273, p282, and p302 phospho-band intensities normalized to total protein (e.g., p273/total cMyBPC) were expressed as a percentage of PKA^+^-treated phosphorylation. Samples from 4 mice per group were obtained in the dobutamine infusion experiment for representative Western blots of p273, p282, and p302 phosphorylation. To determine cTnT and cTnI expression and phosphorylation, a separate set of gels was stained with Pro-Q diamond phosphoprotein stain (Invitrogen) and Coomassie blue. The relative phosphorylation between different groups was determined by the intensity of the Pro-Q band normalized to the Coomassie band intensity. Densitometric scanning of Western blots and stained gels was performed using ImageJ (61).

### IHC staining and fluorescence imaging.

Immunofluorescent determination of cMyBPC and C0C2 fragment localization in myofibrils was performed as previously described (63). Briefly, 2-mm cubes of flash frozen ventricular tissue were thawed in relaxing buffer at 4°C and then placed in rigor buffer for homogenization. Myofibrils were skinned using 1% Triton X-100, centrifuged at 1500*g* for 10 minutes, and resuspended in fresh rigor buffer containing BSA (1 μg/mL) to prevent clumping.

IHC staining of freshly made myofibril preparations was carried out at ~25°C. A total of 25 μL of myofibril samples was dispersed on microscope slides and allowed to partially dry before fixing for 10 minutes with the addition of 35 μL of 4% PFA. Each sample was washed with PBS 3 times and then incubated with 50 μL of 5% goat serum for 30 minutes for blocking. A total of 50 μL of primary antibodies (Santa Cruz Biotechnology Inc., cMyBPC antibody, 1:1000, catalog sc-137237; MilliporeSigma, α-actinin antibody, 1:100, catalog SAB4503474) was added for 1-hour incubation. After washing with PBS 3 times, samples were incubated with secondary antibody (Invitrogen, goat anti–mouse Alexa Fluor 488, 1:500, catalog A-21121; goat anti–rabbit Texas red, 1:500, catalog T2767) for 30 minutes. After washing samples with PBS 3 times, Prolong Gold antifade reagent with DAPI (Invitrogen) was used as mountant and allowed to cure overnight. Samples were imaged using an Olympus Fluoview-1000 confocal microscope.

### Experimental protocols in skinned myocardium experiments.

Steady-state and XB kinetic measurements in skinned ventricular preparations were performed as described previously (35). Briefly, frozen ventricular tissue was homogenized in a relaxing solution to obtain multicellular ventricular preparations, which were then detergent skinned using 1% Triton X-100 for 1 hour. Multicellular ventricular preparations with dimension of approximately 100 × 400 μm (width by length) were selected for experiments. Ca^2+^ solutions contained the following (in mM): 14.5 creatine phosphate, 7 EGTA, and 20 Imidazole. Additionally, the maximal activating solution (pCa 4.5; pCa = –log [Ca^2+^]_free_) contained 65.45 KCl, 7.01 CaCl_2_, 5.27 MgCl_2_, and 4.81 ATP, whereas the relaxing solution (pCa 9.0) contained 72.45 KCl, 0.02 CaCl_2_, 5.42 MgCl_2_, and 4.76 ATP. The pH of the Ca^2+^ solutions was set to 7.0 with KOH, and the ionic strength was 180 mM. A series of submaximal pCa solutions, containing varying amounts of [Ca^2+^]_free_, were then prepared by combining appropriate volumes of pCa 9.0 and 4.5 stock solutions, and experiments were conducted at ~25°C ± 1°C.

Skinned multicellular ventricular preparations were securely attached between a motor arm (315C; Aurora Scientific Inc.) and a force transducer (403A; Aurora Scientific Inc.), as described previously (64, 65). Changes in motor arm positions and force transducer signals were sampled at 2000 Hz using a custom-built SL control software (66). For all mechanical measurements, the SL of ventricular preparations was set to 2.1 μm (38). Force-pCa relationships were determined by measuring the forces generated by skinned myocardial preparations isolated from WT and AAV9-treated cMyBPC^–/–^ hearts in a series of pCa solutions that generate submaximal to maximal forces. The apparent cooperativity of force generation was estimated from the steepness of Hill plot transformation of the force-pCa relationships (67). The force-pCa data were fit using the equation P/P_o_ = [Ca^2+^]**^nH^/(*k*^nH^ + [Ca^2+^]**^nH^), where *n*_H_ is the Hill coefficient and *k* is the pCa needed to elicit half-maximal force (pCa_50_) (68).

The stretch-activation protocol used in this study was performed as previously described (64, 69). Skinned myocardial preparations were activated in a pCa solution that generated submaximal forces at pCa 6.1 and pCa 6.0. Once the myocardial preparations attained a steady-state force, they were subjected to a sudden 2% stretch of their initial muscle length (ML), held at the new ML for 8 seconds, and returned back to their initial ML. The characteristic features of the cardiac muscle stretch activation responses have been described earlier (68, 70). Briefly, a sudden 2% stretch in ML elicits an instantaneous spike in the force (P1) due to a sudden strain of elastic elements within the strongly bound XBs (Phase 1). Force then quickly falls (phase 2) to a minimum, due to the detachment of strained XBs into a non–force bearing pool, with a dynamic rate constant *k*_rel_ (an index of XB detachment from actin). The lowest point of phase 2 (nadir) is indicated by P2 which denotes the magnitude of XB detachment. Following the nadir, there is a gradual force rise (phase 3), with a dynamic rate constant *k*_df_ (an index of XB recruitment to actin), which happens due to stretch-induced recruitment of additional XBs into the force-bearing state (65). Stretch activation amplitudes were normalized to prestretch steady-state Ca^2+^-activated force as done before (64, 65, 68). The magnitude of new steady-state force (P3) was measured from the prestretch steady-state force to the peak force value attained in phase 3, and P2 was measured from prestretch steady-state force to the nadir of force response in phase 2, while P_df_ was measured as the difference between P3 and P2 values (64, 65, 68).

The *k*_rel_ was measured by fitting a single exponential equation to the time course of force decay using the equation: F(t) = a(–1 + exp[–*k*_rel_ × t]) where “a” is the amplitude of the single exponential phase and *k*_rel_ is the rate constant of the force decay, as described earlier (64, 68). The *k*_df_ represents the rate of recruitment of all XBs that contribute to the delayed force rise following the sudden 2% stretch in ML and was estimated by linear transformation of the half-time of force redevelopment (64, 68) — *k*_df_ = 0.693/t_1/2_, where t_1/2_ is the time (in milliseconds) taken from the nadir (i.e., the point of force redevelopment at the end of phase 2) to the point of half-maximal force in phase 3 of the force response, where maximal force is indicated by a plateau region of phase 3 (i.e., P3) (64, 68).

### Production of exogenous C0C2 protein.

pET-30a(+) vectors containing DNA encoding C-terminal 6× His-tagged mouse C0C2 was obtained from GeneScript and expressed in *E*. *coli* BL21 Star (DE3). Cells were grown in 1 L LB media until OD_600 nm_ reached 0.8–1.0 at 37°C. The culture was cooled on ice for 30 minutes and incubated with shaking at 16°C for 16 hours after induction with 0.5 mM IPTG. Cell pellets were resuspended in 30 mL of cold resuspension buffer (20 mM HEPES [pH 7.5], 500 mM KCl, 10 mM Imidazole, 1 mM AEBSF, and protease inhibitor tablet; cOmpleteULTRA, Roche), flash frozen in liquid N_2_, and stored at –80°C until purification.

Purification was performed as previously described (71), with the following modifications. Briefly, pellets were thawed and then lysed using an Avestin Emulsiflex C3 homogenizer (ATA Scientific). Clarified lysates were rocked for 2 hours with Ni-NTA resin at 4°C. C0C2 protein was eluted from resin by gravity flow in a fritted column using 3 mL of elution buffer (20 mM HEPES [pH 7.5], 500 mM KCl, 250 mM Imidazole). DTT was added to the eluate to a final concentration of 1 mM. Eluate was then concentrated using a centrifugal concentrator with a 30 kDa molecular weight cut off (Pall) to 500 μL before being passed over an Enrich SEC 70, 10 × 300 mm column (Bio-Rad) equilibrated in rigor buffer. Fractions containing C0C2 were collected (Supplemental Figure 3A) and protein concentration was determined by UV absorbance at 280 nm. Confirmation of C0C2 was performed using SDS-PAGE and Western blot analysis as described above. Protein purity was analyzed by SDS-PAGE and Coomassie staining and found to be > 95% (Supplemental Figure 3B).

### Statistics.

Data were expressed as mean ± SEM. Statistical comparisons between 2 groups were made using a 2-tailed *t* test. Statistical comparisons between 3 or more groups were made using a 1-way ANOVA followed by post hoc Tukey’s multiple comparisons test. All statistical analyses were performed using GraphPad Prism version 6.01 for Windows (GraphPad Software, www.graphpad.com). *P* < 0.05 was considered statistically significant.

### Study approval.

All procedures involving animal care and handling, including viral injections and BSL-1/ABSL-1 practices, were reviewed and approved by the Case Western Reserve University Animal Care and Use Committee.

## Author contributions

JL and JES conceived and designed the experiments. JL, RM, CYD, and JBH conducted experiments and analyzed data. NB and RR provided the exogenous C0C2 and commented on the study design. JL and JES wrote the manuscript. All authors commented on the manuscript.

## Supplementary Material

Supplemental data

## Figures and Tables

**Table 1:**
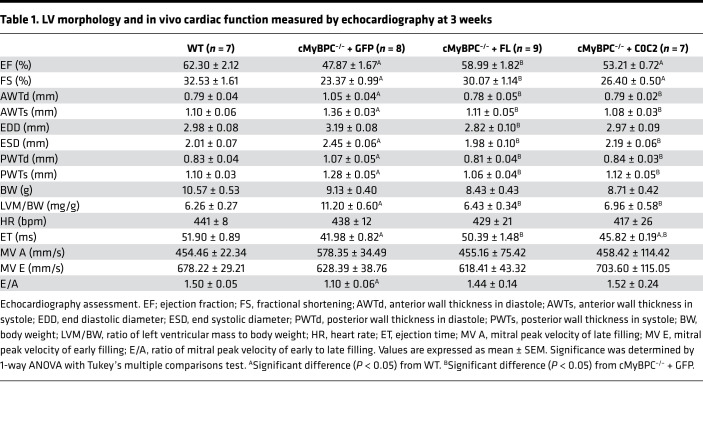
LV morphology and in vivo cardiac function measured by echocardiography at 3 weeks

**Table 2:**
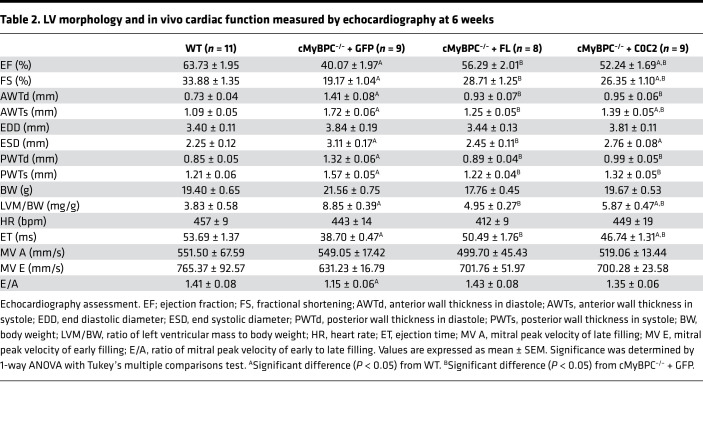
LV morphology and in vivo cardiac function measured by echocardiography at 6 weeks

**Table 3:**
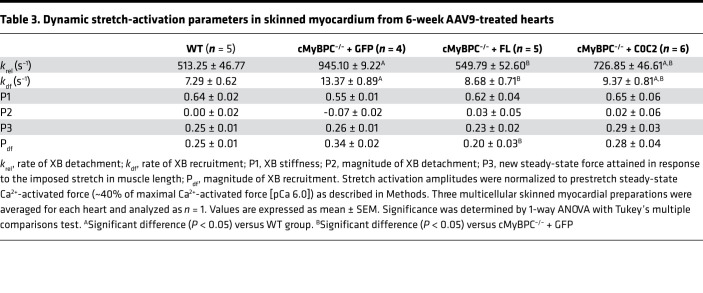
Dynamic stretch-activation parameters in skinned myocardium from 6-week AAV9-treated hearts

**Table 4:**
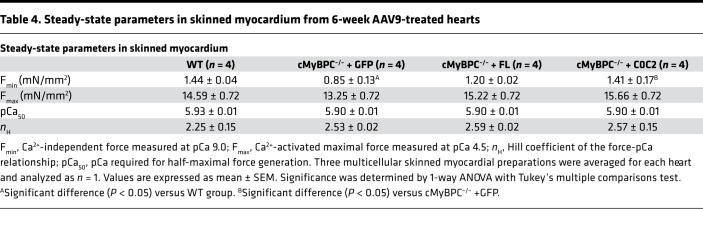
Steady-state parameters in skinned myocardium from 6-week AAV9-treated hearts

## Version 1 08/04/2020

In-Press Preview

## Version 2 09/03/2020

Electronic publication
