# Supplementary material for: AAV9 gene transfer of cMyBPC N-terminal domains ameliorates cardiomyopathy in cMyBPC-deficient mice
Source: JCI Insight. 2020 Sep 3;5(17):e130182. doi: 10.1172/jci.insight.130182 (PMC7526450; doi:10.1172/jci.insight.130182)
Supplement: Supplemental data [file jciinsight-5-130182-s047.pdf]

Supplemental Document

Table S1. Effect of exogenous C0C2 on steady-state parameters in skinned myocardium

Steady-state parameters in skinned myocardium		
	WT (n=4)	cMyBPC$^{-/-}$ (n=4)
F_{min} (mN/mm^2) baseline	1.01±0.16	0.85±0.13
F_{min} (mN/mm^2) +C0C2	1.17±0.16	1.70±0.34*
F_{max} (mN/mm^2) baseline	16.07±1.36	15.28±3.01
F_{max} (mN/mm^2) +C0C2	16.71±1.63	15.60±2.86

Quantification of Ca^{2+}-independent force (F_{min}) in pCa 9.0 and maximum Ca^{2+} activated force (F_{max}) in pCa 4.5, before and after a 10 minute 1.0µM C0C2 incubation. F_{min}: Ca^{2+}-independent force measured at pCa 9.0; F_{max}: Ca^{2+}-activated maximal force measured at pCa 4.5. n=number of hearts per group. Values are expressed as mean ± S.E.M; * indicates significant difference ($p<0.05$) by paired t-test when comparing respective pre- and post- C0C2 incubation.

Table S2. Effect of exogenous C0C2 on stretch-activation parameters in skinned myocardium

Dynamic stretch-activation parameters in skinned myocardium		
	WT (n=4)	cMyBPC$^{-/-}$ (n=4)
k_{rel} (s^{-1}) baseline	481.16±68.95	947.72±41.85
k_{rel} (s^{-1}) +C0C2	459.12±69.74	733.64±50.37*
Δ k_{rel} (%)	-4.84±4.47	-22.44±5.14
k_{df} (s^{-1}) baseline	3.35±0.53	7.07±0.28
k_{df} (s^{-1}) +C0C2	3.21±0.46	4.60±0.39*
Δ k_{df} (%)	-3.89±3.81	-33.90±7.95#

Stretch-activation parameters measured before and after a 10 minute 1.0µM C0C2 incubation at pCa 6.1. k_{rel}: rate of XB detachment; k_{df}: rate of XB recruitment. n=number of hearts per group. Values are expressed as mean ± S.E.M; * indicates significant difference ($p<0.05$) by paired t-test when comparing respective pre- and post- C0C2 incubation. # indicates significant difference in % change of stretch-activation rate constant between WT and cMyBPC$^{-/-}$ groups.

Figure S1

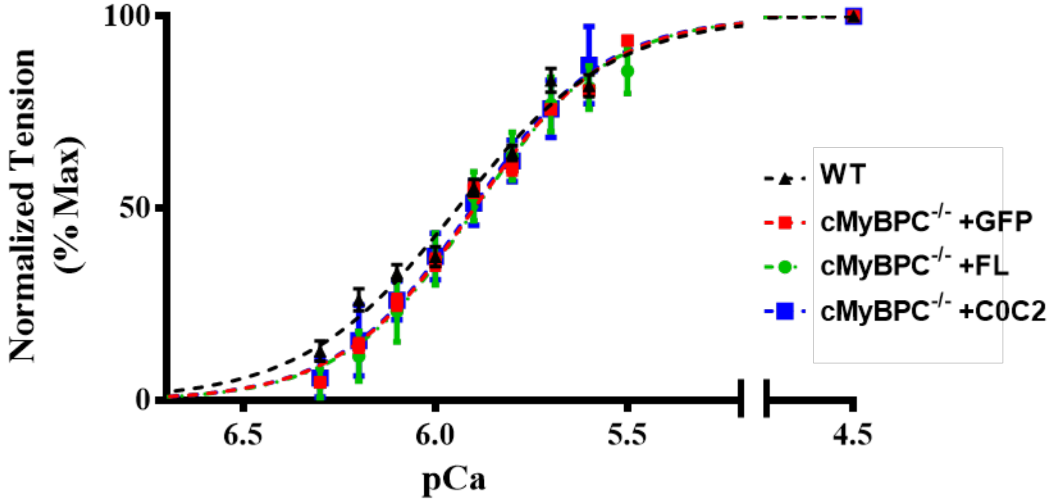

Figure S1: Myofilament Ca^{2+} sensitivity (pCa_{50}) in cardiac preparations from AAV-9 injected groups. Force-pCa relationships were constructed by plotting normalized forces generated at a range of pCa to assess pCa_{50} in WT hearts and $cMyBPC^{-/-}$ hearts injected with AAV9-GFP, or -FL, or –C0C2 cMyBPC. No significant differences in pCa_{50} were observed between the groups. 12 myocardial preparations from 4 hearts were used for all the groups.

Figure S2:

```
  1  atgccggagc cagggaagaa accagtgtca gccttcaaca agaagccaag
 51  gtcagcggag gtgaccgctg gcagtgctgc cgtgttcgag gctgAgacgg
101  agcggtcagg cgtgaaggtg cggtggcagc gggatggcag cgacatcacc
151  gccaatgaca agtatggttt ggcagcagag ggcaagcgGc acacactgac
201  agtgcgggat gcgagccctg atgaccaggg ttcctacgcg gtcattgcag
251  gctcctcaaa ggtcaagttt gacctcaagg tcacagagcc agcccctcca
301  gagaaggcag aatctgaagt tgctccagga gcccccaaag aagtccctgc
351  tccagccact gagttggaag aaagtgtctc aagtcctgaa gggtcagtct
401  cggtaaccca ggatggctca gctgcagagc atcagggagc ccctgatgac
451  cctattggcc tctttctgat gcgaccacag gatggtgagg tgaccgtggg
501  cggcagcatt gtcttctcag cccgagtggc tggggccagc ctcctgaaac
551  cgcctgtggt caagtggttc aagggcaagt gggtggacct gagcagcaaa
601  gtgggccagc acTtgcagct gcatgacagc tatgacagag ccagcaaggt
651  ctacttgttt gagttgcaca tcacagatgc tcagaccact tctgctgggg
701  gctaccgctg tgaggtgtct accaaggaca aatttgacag ctgtaActtc
751  aacctcactg tccatgaggc cattggttct ggagacctgg acctcagatc
```

801 agctttccga cgcacgAGCc tGgcgggagc AggTcggaga accAGtgaca

851 gccatgaaga tgctgggact ctggacttta gttccctgct gaagaagaga

901 gacAGTttcc ggagggactc aaagctggag gcacctgctg aagaagacgt

951 gtgggagatc ctgagacagg caccgccgtc agaatatgag cgcatcgcct

1001 tccagcacgg agtcacagac cttcgaggca tgctgaagag gctcaagggc

1051 atgaagcagg atgaaaagaa gagcacagcc tttcagaaga agctggagcc

1101 tgcctaccag gtaaacaagg gccacaagat tcggcttact gtggaactgg

1151 ctgatccgga cgccgaagtc aagtggctta agaatggaca ggagatccag

1201 atgagtggca gcaagtacat cttcgagtcc gtcggtgcca agcgcaccct

1251 gaccatcagc cagtgctcac tggctgacga cgcagcctac cagtgtgtgg

1301 tgggggcga gaagtgcagc acggagctct ttgtcaaaga gcccccggtg

1351 ctgatcactc ggtccctgga agaccagctg gtgatggtgg gtcagcgggt

1401 ggagtttgag tgtgaggtct cagaagaagg ggcccaagtc aaatggctga

1451 aggatggggt tgaGctgaca cgtgaggaga ccttcaaata ccggttcaag

1501 aaagatgggc ggaaacacca cttgatcatc aatgaagcaa ccctggagga

1551 tgcaggacac tatgcagtac gcacaagtgg aggccagtca ctggctgagc

1601 tcattgtgca agagaagaag ttggaggtat accaaagcat cgcggacctg

1651 gcagtgggag ccaaggacca ggctgtgttt aagtgtgagg tttcagatga

1701 gaatgtacgc ggcgtgtggc tgaagaatgg gaaggaactg gtgcctgaca

1751 accgcataaa ggtgtcccat ataggccggg tccacaaact gaccattgac

1801 gatgtcacac ctgctgatga ggctgactac agctttgtcc ctgaagggtt

1851 tgcctgcaac ctgtctgcca agctccactt catggaggtc aagattgact

1901 ttgtgcctag gcaggaacct cccaagatcc acttggattg tcccggcagc

1951 acaccagaca ccattgtggt tgttgctggg aacaagttac gcctggatgt

2001 ccctatttct ggagaccctg ctcccactgt ggtctggcag aagactgtaa

2051 cacaggggaa gaaggcctca actgggccac accctgatgc cccagaagat

2101 gctggtgctg atgaggagtg ggtgtttgat aagaagctgt tgtgtgagac

2151 tgagggccgg gtccgggtgg agaccaccaa agaccgcagc gtctttacag

2201 tcgaaggggc agAgaaggaa gatgaaggtg tctacacagt cacagtaaag

2251 aaccccgtgg gcgaggacca ggtcaacctc acagtcaagg tcatcgatgt

2301 cccagatgct cctgcggccc ctaagatcag caacgtgggc gaggactcct

2351 gcactgtgca gtgggaaccg cctgcctatg atggcgggca gccggtcctg

2401 ggatacatcc tggagcgcaa gaagaaaaag agctacaggt ggatgaggct

2451 caactttgat ctgctgcggg agctgagcca cgaggcgagg cgcatgatcg

2501 agggtgtagc ctatgagatg cgagtctacg cagtcaatgc cgtgggaatg

2551 tccaggccca gccctgcctc tcagcccttc atgcctattg ggccccctgg

2601 cgaaccaacc cacttggctg tggaggatgt gtcagacacc actgtctcac

2651 tcaagtggcg gcccccagag cgcgtggggg ccggtggcct ggacggatac

2701 agcgtggagt actgccagga gggatgctcc gagtggacac ctgctctgca

2751 ggggctgaca gagcgcacat cgatgctggt gaaggaccta cccactgggg

2801 cacggctgct gttccgagta cgggcacaca atgtggcagg tcctggaggc

2851 cctatcgtca ccaaggagcc tgtgacagtg caggagatac tgcaacgacc

2901 acggctccaa ctgcccagac acctgcgcca gaccatccag aagaaagttg

2951 gggagcctgt gaacctcctc atccctttcc agggcaaacc ccggcctcag

3001 gtgacctgga ccaaagaggg gcagcccctg gcaggtgagg aggtgagcat

3051 ccggaacagc cccacagaca cgatcttgtt catccgagct gcccgccgca

3101 cccactcggg cacctaccag gtgacagttc gcattgagaa catggaggac

3151 aaggcaacgc tgatcctgca gattgtggac aagccaagtc ctccccagga

3201 tatccggatc gttgagactt ggggtttcaa tgtggctctg gagtggaagc

3251 caccccaaga tgatggcaat acagagatct ggggttatac tgtacagaaa

3301 gctgacaaga agaccatgga gtggttcacg gttttggaac actaccgacg

3351 cactcactgt gtggtatcag agcttatcat tggcaatggc tactacttcc

3401 gggtcttcag ccataacatg gtgggttcca gtgacaaagc tgccgccacc

3451 aaggagccag tctttattcc aagaccaggc atcacatatg agccacccaa

3501 atacaaggcc ctggacttct ctgaggcccc aagcttcacc cagcccttgg

3551 caaatcgctc catcattgca ggctataatg ccatcctctg ctgtgctgtc

3601 cgaggtagtc ctaagcccaa gatttcctgg ttcaagaatg gcctggatct

3651 gggagaagat gctcgcttcc gcatgttctg caagcaggga gtattgaccc

3701 tggagatcag gaaaccctgc ccctatgatg gtggtgtcta tgtctgcagg

3751 gccaccaact tgcagggcga ggcacagtgt gagtgccgcc tggaggtgcg

3801 agttcctcag tga

Figure S2: cMyBPC cDNA sequence. Murine FL cMyBPC cDNA Sequence used in AAV9-vectored constructs. The N-terminal C0C2 sequence is highlighted in grey.

Figure S3

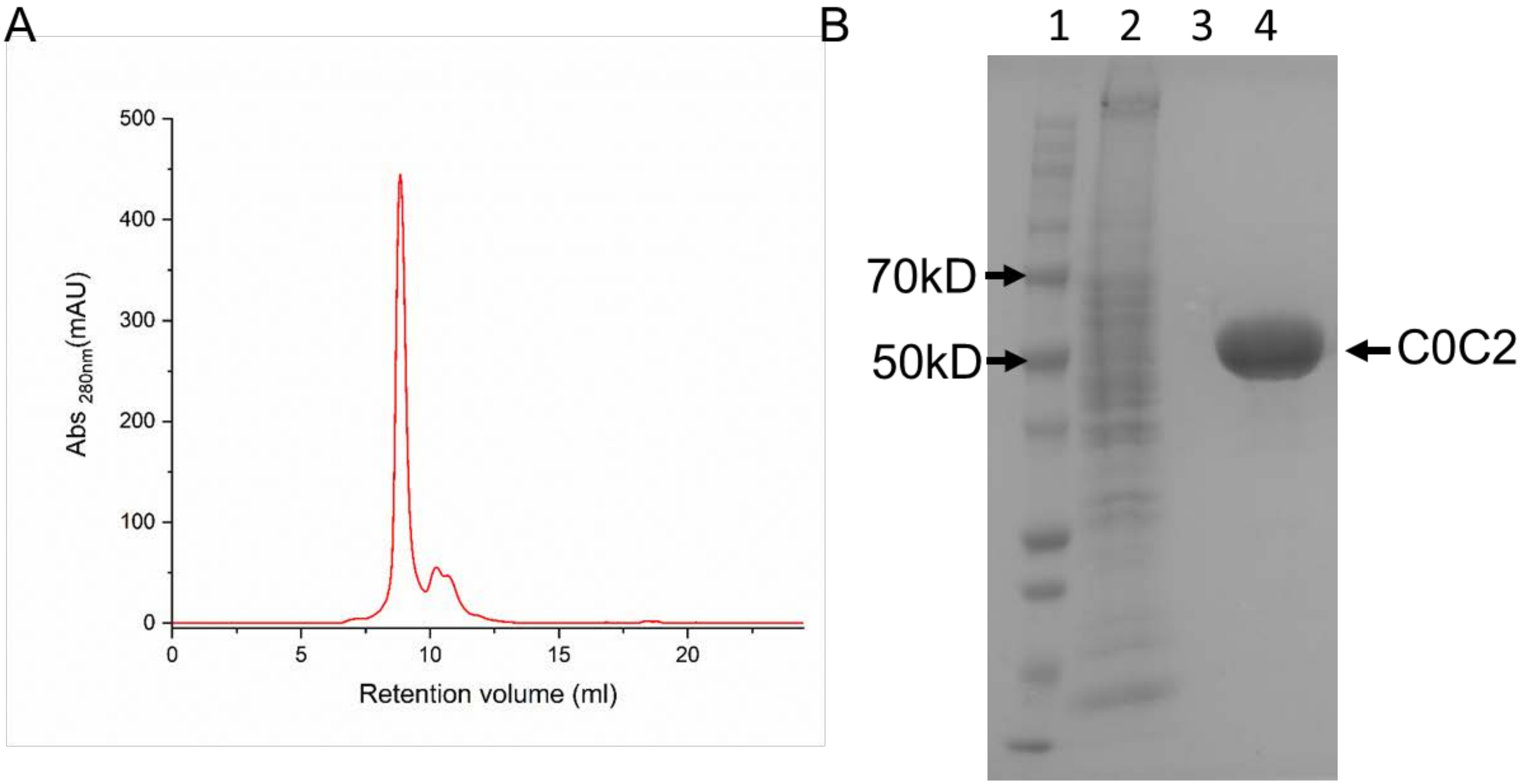

Figure S3: Recombinant C0C2 purification. (A) Representative size-exclusion chromatography (SEC) profile of recombinant C0C2 purification. A single major peak is found at the elution volume corresponding to monomeric C0C2 (~49kD). **(B)** Representative Coomassie staining image of SDS-page gel with molecular weight ladder (lane 1), total protein lysate before purification (lane 2), and protein sample from peak fraction collected from SEC (lane 4). Lane 3 was left blank to allow for SEC sample to run unimpeded. The higher concentration of lane 3 loading confirms the absence of major trace contaminates after purification.

Figure S4

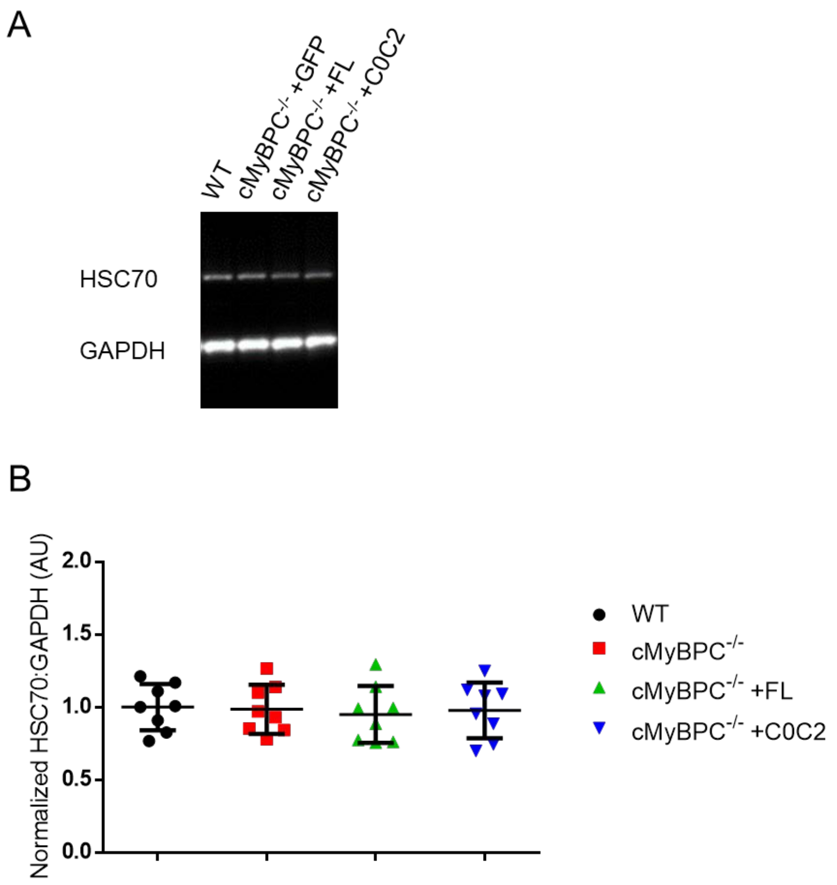

Figure S4: Quantification of relative HSC70 expression. (A) Representative Western blot showing the expression of HSC70 and GAPDH in WT (lane 1), AAV9-GFP treated cMyBPC$^{-/-}$ (lane 2), AAV9-FL treated cMyBPC$^{-/-}$ hearts (lane 3), and AAV9-C0C2 treated cMyBPC$^{-/-}$ hearts (lane 4). (B) Quantification of HSC70:GAPDH in WT, AAV9-GFP treated cMyBPC$^{-/-}$, AAV9-FL treated cMyBPC$^{-/-}$ hearts, and AAV9-C0C2 treated cMyBPC$^{-/-}$ hearts. n=8 per group.
